# Inhibition of Matrix Metalloproteinases and Cancer Cell Detachment by Ru(II) Polypyridyl Complexes Containing 4,7-Diphenyl-1,10-phenanthroline Ligands—New Candidates for Antimetastatic Agents

**DOI:** 10.3390/ph14101014

**Published:** 2021-10-01

**Authors:** Przemysław Gajda-Morszewski, Ilona Gurgul, Ewelina Janczy-Cempa, Olga Mazuryk, Michał Łomzik, Małgorzata Brindell

**Affiliations:** 1 Faculty of Chemistry, Jagiellonian University in Krakow, Gronostajowa 2, 30-387 Krakow, Poland; pgmorszewski@doctoral.uj.edu.pl (P.G.-M.); ilona.gurgul@doctoral.uj.edu.pl (I.G.); ewelina.janczy@doctoral.uj.edu.pl (E.J.-C.); olga.mazuryk@uj.edu.pl (O.M.); michal.lomzik@chemia.uni.lodz.pl (M.Ł.); 2 Department of Organic Chemistry, Faculty of Chemistry, University of Łódź, ul. Tamka 12, 91-403 Łódź, Poland

**Keywords:** ruthenium(II) polypyridyl complexes, matrix metalloproteinases, inhibition of MMP-2 and MMP-9, cell adhesion properties, cytotoxicity, cell detachment, cellular uptake

## Abstract

Primary tumor targeting is the dominant approach in drug development, while metastasis is the leading cause of cancer death. Therefore, in addition to the cytotoxic activity of a series of Ru(II) polypyridyl complexes of the type [Ru(dip)_2_L]^2+^ (dip: 4,7-diphenyl-1,10-phenanthroline while L = dip; bpy: 2,2′-bipyridine; bpy-SC: bipyridine derivative bearing a semicarbazone 2-formylopyridine moiety; dpq, dpq(CH_3_)_2_, dpb: quinoxaline derivatives) their ability to inhibit cell detachment was investigated. In vitro studies performed on lung cancer A549 cells showed that they accumulate in cells very well and exhibit moderate cytotoxicity with IC_50_ ranging from 4 to 13 µM. Three of the studied compounds that have dip, bpy-SC, or dpb ligands after treatment of the cells with a non-toxic dose (<^1^/_2_IC_50_) enhanced their adhesion properties demonstrated by lower detachment in the trypsin resistance assay. The same complexes inhibited both MMP-2 and MMP-9 enzyme activities with IC_50_ ranging from 2 to 12 µM; however, the MMP-9 inhibition was stronger. More detailed studies for [Ru(dip)_2_(bpy-SC)]^2+^, which induced the greatest increase in cell adhesion, revealed that it is predominately accumulated in the cytoskeletal fraction of A549 cells. Moreover, cells treated with this compound showed the localization of MMP-9 to a greater extent also in the cytoskeleton. Taken together, our results indicate the possibility of a reduction of metastatic cells escaping from the primary lesion to the surrounding tissue by prevention of their detachment and by influencing the activity of MMP-2 and MMP-9.

## 1. Introduction

Cancer is a group of diseases associated with abnormal cells that grow out of control with the potential to invade other parts of the organism. The uncontrolled cell growth may lead to the formation of tissue mass called primary tumor [1]. The process by which cancer cells degrade surrounding tissues, overcome biological barriers, colonize, and proliferate in a distant part of the organism to form a secondary tumor is called metastasis. In numerous types of cancer, metastasis is the most dangerous attribute of diseases, which is responsible for poor prognosis, complications, and high mortality [2,3,4]. Despite increasing understanding of the risks associated with the formation and development of metastasis, still, there are no efficient antimetastatic therapies. Therefore, design agents that would not only target the primary tumor but also inhibit metastasis are of great importance. 

Metastasis is a very complex process involving several steps [5,6] that begins with the detachment of neoplastic cells from the primary tumor. Extracellular matrix (ECM), a three-dimensional non-cellular component present within all tissues that provides structural and biochemical support to surrounding cells, can be regarded as a major obstacle in the first stage of metastasis. It constitutes a physical barrier and basement for cells, and its composition ensures proper ECM-cell and cell–cell interaction and adhesion. Matrix metalloproteinases (MMPs) are enzymes capable of degrading and remodeling ECM [7,8]. They play an important role in cancer cell survival and expansion as they are involved in all stages of carcinogenesis [9]. By degradation of various adhesion molecules, they can modulate cell–cell and cell–ECM adhesion. For metastasis progression matrix metalloproteinases-2 and -9 (MMP-2 and MMP-9) are crucial since they regulate migration and epithelial to mesenchymal transition. The proteolytic activity of these enzymes results in the degradation of physical barriers enabling cancer cell invasion. In addition, they are involved in tumor angiogenesis and vasculogenesis. Considering such a diverse role of MMP-2 and MMP-9, targeting these enzymes seems to be a good strategy to achieve antimetastatic activity.

Ruthenium polypyridyl complexes have been tested for many years for their use in anticancer therapy as cytotoxic agents [10,11,12]. Recently, ours and other studies have shown that ruthenium polypyridyl compounds in addition to their well-documented cytotoxic activity can affect various cell properties such as detachment, motility, invasion, colonization ability, and others that are crucial for metastasis formation and development [13,14,15,16,17,18,19]. The detailed mechanism of such activity is still largely unknown; however, some of these compounds have been shown to strongly alter cell adhesion properties. MMP-2 and MMP-9 were considered among the postulated targets, and it was shown that some of the ruthenium polypyridyl complexes may inhibit their activity [19], while others may down-regulate their secretion [14,16]. In this study, for the first time, we investigate the relationship between the localization of Ru in the cell and the expression level of MMPs by analyzing subcellular fractions, which may strengthen the hypothesis of MMPs as potential targets for Ru polypyridyl complexes. 

**Figure 1:**
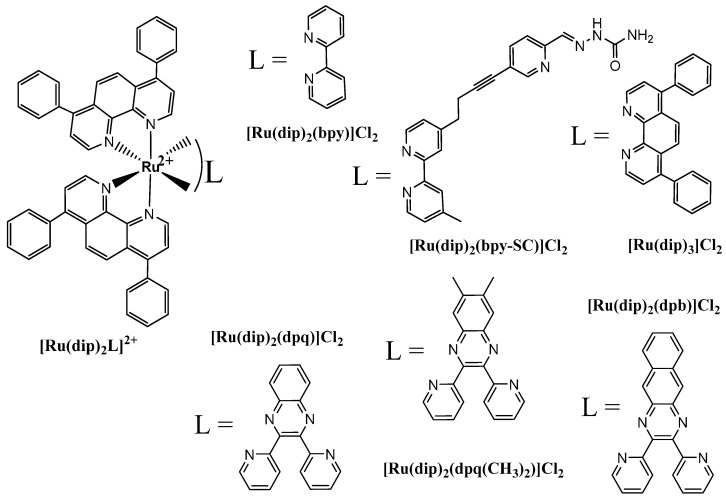
The studied ruthenium(II) polypyridyl complexes.

In this work, a series of Ru(II) polypyridyl complexes of the type [Ru(dip)_2_L]^2+^ (L = bpy, bpy-SC, dip, dpb, dpq, or dpq(CH_3_)_2_; depicted in Figure 1) was investigated to gain insight into their effect on cell adhesion properties as well as their potency in matrix metalloproteinases inhibition. The impact of the studied compounds on human lung adenocarcinoma A549 cells viability and their susceptibility to detachment was assessed and related to their uptake and localization. Moreover, their influence on the MMP-2 and MMP-9 expression in A549 cells and inhibition potency towards these enzymes were evaluated. The *trans*-[tetrachlorido(1H-imidazole)(S-dimethylsulphoxide)ruthenate(III)] known as NAMI-A with well-documented antimetastatic properties and the broad-spectrum MMP inhibitor GM0006 were used as reference compounds. NAMI-A is an unquestionable leader in research on antimetastatic properties of Ru complexes, and detailed information on its activity is given in recent reviews [20,21].

The study was designed to recognize the role of L ligand in the observed biological activity and examine the relationship between hampering of cell detachment and decreasing activity of MMP-2 and MMP-9 either by reducing their expression or by direct inhibition.

## 2. Results and Discussion 

### 2.1. Cytotoxicity and Uptake

**Figure 2:**
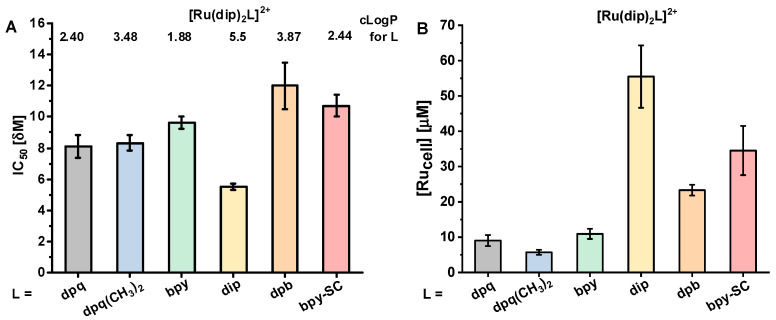
(**A**) Cytotoxicity (IC_50_) of the studied Ru(II) complexes evaluated for A549 cells along with the calculated lipophilicity (clogP) of the ligand L in complexes of the type [Ru(dip)_2_L]^2+^ with the use of ChemDraw Professional 17.1. (**B**) The total amount of the accumulated ruthenium ions in A549 cells was determined after 24 h incubation with 1 µM of Ru(II) complexes presented as a concentration in single cells.

The viability of human lung adenocarcinoma cells (A549) decreased by treatment with all studied Ru(II) polypyridyl complexes in a dose-dependent manner. The cytotoxicity of the studied compounds ranged from 5 to 12 µM (Figure 2), which makes them moderately cytotoxic. However, they are much more cytotoxic than cisplatin, with IC_50_ of 70 µM [22]. All studied complexes accumulated in live A549 cells. Quantification of the ruthenium ion concentration using ICP-MS revealed that treatment of cells with 1 µM concentration of Ru(II) compounds increased their concentration in a cell by 6 to 55-fold, depending on compound (Figure 2B). The most cytotoxic compound [Ru(dip)_3_]^2+^ was characterized by the highest uptake by cells, which correlated well with the highest lipophilicity. There is no direct correlation between the lipophilicity, uptake, and cytotoxicity for the other tested compounds, suggesting different accumulation profiles and targets, which are determined by the structure of the L ligand. Therefore, knowledge of complex lipophilicity does not allow for the prediction of the degree of cellular uptake. Furthermore, not only the amount of the accumulated compound is important, but also its localization and activity towards potential targets, most likely different proteins. 

### 2.2. Effect on Adherence Properties of Cells 

**Figure 3:**
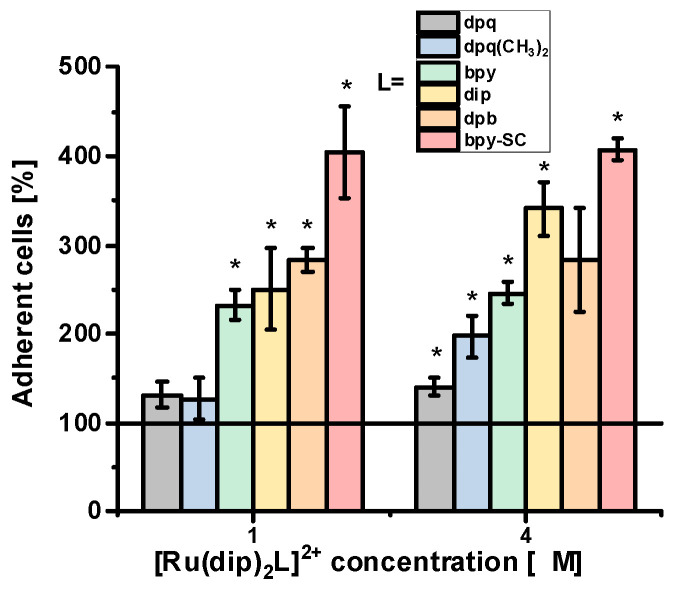
A549 cell adherence, evaluated as the percentage of remaining adherent cells upon controlled trypsin treatment compared to control after incubation with the studied Ru(II) complexes. for 24 h. Untreated cells were used as a control (100%). * *p* < 0.05.

Change in cell adherence upon treatment with Ru(II) polypyridyl complexes was evaluated as the percentage of the remaining adherent cells upon controlled trypsin treatment compared to control. As shown in Figure 3, the highest increase in cell adherence even up to 400% vs. control was observed for Ru complex with bpy-SC ligand. This complex has already been described as a very efficient agent also in reducing the detachment of human pancreatic cancer cells [19]. The other three complexes with bpy, dip, or dpb ligands also significantly enhanced the adhesive properties of A549 cells, while those with dpq and dpq(CH_3_)_2_ ligand had a marginal effect on cells adhesion. The observed effect was not concentration-dependent and already detected for as low concentration of compounds as 1 µM, which is far away from IC_50_ for all studied compounds. Considering the lipophilicity of ligand L and the cytotoxicity of the Ru complexes, any direct correlation can be found that points to the structure of the L ligand as a critical factor affecting the adhesion properties of cells. Treatment of A549 cells with NAMI-A resulted also in a significant increase of the adhesion strength of cells, up to ca. 250% compared to control but it was observed at a much higher concentration of 100 µM (still non-toxic). A similar effect of NAMI-A was already reported for B16F10 melanoma cells which treatment with 100 µM of compound resulted in a significant increase of the adhesion strength, even up to 162% vs. control [23]. In contrast, upon treatment of cells with GM6001, a broad-spectrum metalloproteinase inhibitor, in a concentration range 1–8 µM (non-toxic concentration) no alteration in cell adhesion properties was observed.

**Figure 4:**
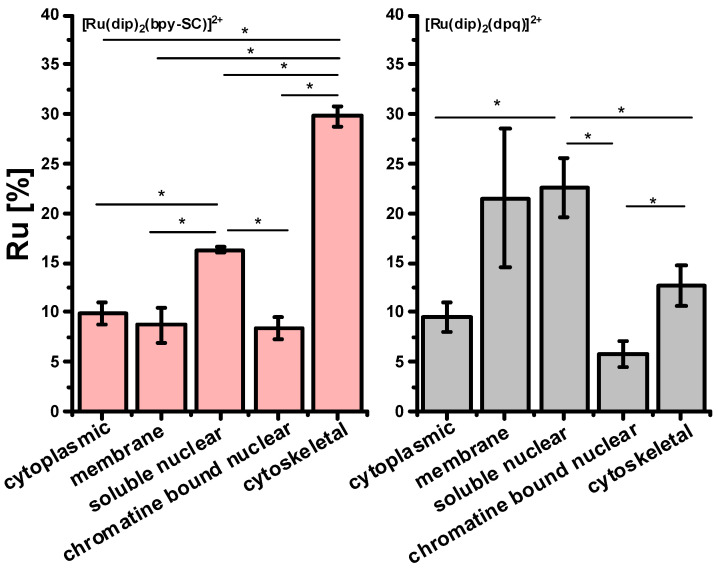
Ruthenium accumulation profile in different cell compartments after incubating A549 cells for 24 h with 4 µM of [Ru(dip)_2_(bpy-SC)]^2+^ or [Ru(dip)_2_(dpq)]^2+^. * *p* < 0.05.

The localization of the accumulated Ru(II) polypyridyl complexes in the cell depends largely on their ligands and occurs in various organelles, the cytoplasm, or it can be dispersed throughout the cell [12]. Our previous studies revealed that Ru compounds containing two dip ligands localized primarily in mitochondria and the endoplasmic reticulum [22,24] while [Ru(dip)_3_]^2+^ was shown to be mainly localized in the lysosome and the mitochondria [25]. To determine whether the localization of the studied Ru(II) polypyridyl complexes could be correlated with their effect on cell resistance to trypsin, the accumulation of Ru in different cellular compartments was measured for complexes weakly ([Ru(dip)_2_(dpq)]^2+^) or strongly ([Ru(dip)_2_(bpy-SC)]^2+^) increasing cell adherence. As shown in Figure 4 ruthenium ions were present in each cellular fraction. For both compounds, a large amount of ruthenium ions was observed in the nuclear fraction. However, [Ru(dip)_2_(bpy-SC)]^2+^ accumulated to a much greater extent in the cytoskeleton fraction, while [Ru(dip)_2_(dpq)]^2+^ was primarily localized in membrane extract (Figure 4). Thus, Ru complex localized in the cytoskeleton fraction of the cell may be account for the observed effect on cell adhesion properties. The focal adhesion is a structural unit that is responsible for cell adhesion to the substrate, in our experiment to the plastic surface of the cell culture plate. There are many proteins associated with focal adhesion among which integrins play a crucial role in regulating cell/substrate interaction. Integrins are attached to the actin microfilament components of the cytoskeleton through linker proteins [26]. The ruthenium complex present in the cytoskeleton fraction may interfere with the signaling mechanism between integrins and the cytoskeleton assembly, leading to a positive response that increases cell adhesion to the substrate. Further studies are needed to support this hypothesis. 

### 2.3. MMP-2 and MMP-9 Inhibition 

**Figure 5:**
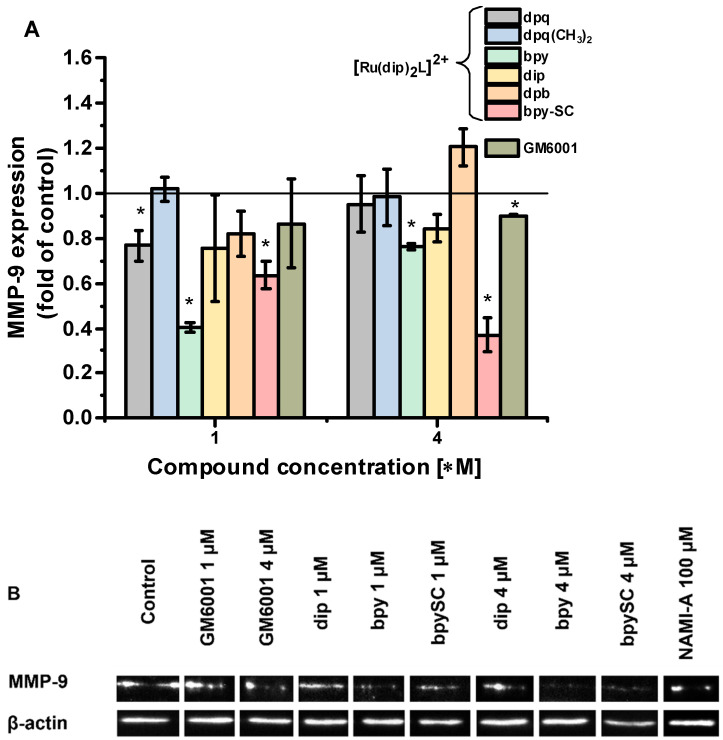
(**A**) The expression levels of the active form of MMP-9 and (**B**) Representative image of Western blot detection of MMP-9 activity measured in A549 cells after 24 h incubation with 1 or 4 µM of Ru complexes or GM6001, and 100 µM of NAMI-A. β-actin was used for normalization. * *p* < 0.05.

MMP-2 and MMP-9 are two key enzymes involved in ECM-degradation processes. It was reported that both enzymes are expressed in A549 cells [27,28]. In this study, the expression of MMP-2 and MMP-9 in Ru-treated cells was assessed by Western blot technique. We were not able to measure MMP-2 activity in A549 cells, as the bands were not detectable. However, for MMP-9 antibody, as shown in Figure 5B and Figure S1, there was a band of high intensity attributed to the active form of the enzyme (approx. 70 kDa). The analysis of intensities of the band attributed to MMP-9 revealed that treatment of A549 cells with [Ru(dip)_2_(bpy-SC)]^2+^ reduced its expression by approx. 36 and 67% after treatment with 1 and 4 µM concentrations, respectively (Figure 5A). The effect of the remaining complexes was not so pronounced and although some of them decreased the expression of MMP-9 (e.g., complexes with bpy or dip ligands), it was not concentration-dependent. A pronounced effect (reduction by 43%) was measured for NAMI-A after treating the cells with a concentration of 100 µM. The in vivo studies have demonstrated that NAMI-A is able to reduce both MMP-9 pro-enzyme and its active form while MMP-2 reduction was less pronounced [23]. Changing the expression levels of MMP-9 is one of, but not the only, mechanisms of biological changes manifested by a decrease in cell detachment ability induced in cells by the investigated Ru(II) polypirydyl complexes.

**Figure 6:**
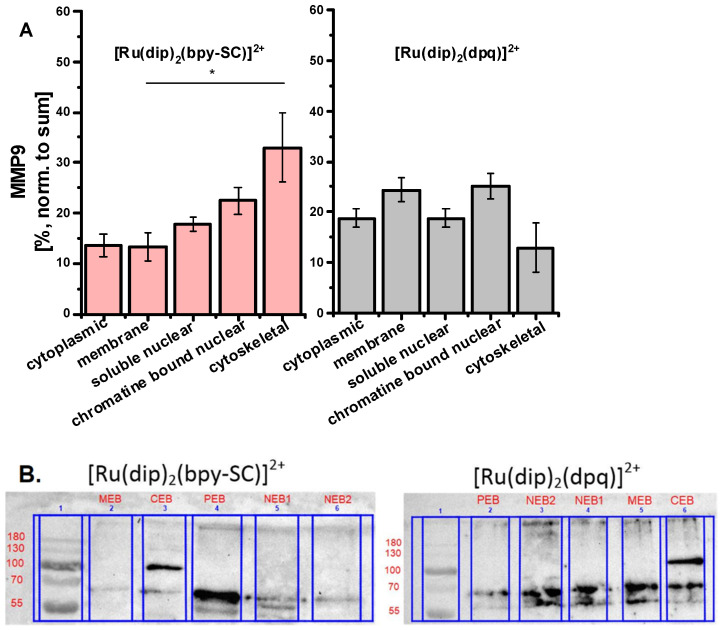
(**A**) MMP-9 localization profile in different cell compartments after incubating A549 cells for 24 h with 4 µM of [Ru(dip)_2_(bpy-SC)]^2+^ or [Ru(dip)_2_(dpq)]^2+^. * *p* < 0.05. (**B**) Representative images of Western blot detection of MMP-9 activity in different cell compartments after incubating A549 cells for 24 h with 4 µM Ru complexes. The following notification for extracts was used: CEB—cytoplasmic, MEB—membrane, NEB1—soluble nuclear, NEB2—chromatin bound nuclear, PEB—cytoskeletal.

In addition, a qualitative evaluation of intracellular localization of MMPs in the prepared subcellular fractions of Ru-treated cells was performed using Western blot. The study was carried out for two complexes: [Ru(dip)_2_(bpy-SC)]^2+^, which showed high potency in inhibiting cell detachment, and for [Ru(dip)_2_(dpq)]^2+^, which displayed a rather weak effect on cell detachment. MMP-2 was not detectable in the prepared extracts, while the presence of MMP-9 was evident. MMP-9 was present to some extent in all the analyzed fractions of Ru-treated cells as shown in Figure 6. Recently, intracellular MMP-9 has been shown to be localized primarily in cytosolic/membrane (70%) and to a significantly lower extent in nuclear compartments (30%) of megakaryocytes cells [29] that is consistent with our observations. For [Ru(dip)_2_(bpy-SC)]^2+^ treated cells higher amount of MMP-9 was observed in the cytoskeletal fraction of A549 cells, while for [Ru(dip)_2_(dpq)]^2+^ membrane fraction was pivotal localization of MMP-9. This suggests that MMP-9 may be one of the molecular targets for [Ru(dip)_2_(bpy-SC)]^2+^, responsible for its influence on the adhesion properties of A549 cells.

The observed colocalization of the expressed MMP-9 and the tested Ru(II) complexes suggests that reported biological activity of Ru complexes may originate, among others, from the inhibition of MMPs activity within the cells. To confirm this, the inhibitory properties of all studied Ru(II) polypyridyl complexes were determined for MMP-9 and additionally for MMP-2 using the fluorogenic substrate FS-6. As shown in Table 1, two complexes, namely [Ru(dip)_3_]^2+^ and [Ru(dip)_2_(bpy-SC)]^2+^, were quite potent in inhibiting both MMP-2 and MMP-9 with IC_50_ of a few micromolar. Most of the studied complexes were better inhibitors against MMP-2 than the reference inhibitor GM6001 except for the complexes containing dpq or dpq(CH_3_)_2_ ligand which did not influence MMPs activity at the studied concentration range. The inhibition of MMP-9 was almost twice as strong as that of MMP-2 for all complexes exhibiting inhibitory activity. NAMI-A showed no inhibitory properties in the studied range of concentration, up to 600 µM.

Interestingly, the strength of inhibition of both MMP-2 and MMP-9 by the studied Ru(II) polypyridyl complexes correlates well with their impact on cell adhesion. Such correlation can be used for further optimizing the structure of ligands in Ru(II) polypyridyl complexes to obtain compounds that will have even higher influence on cell adhesion properties. The compound [Ru(dip)_2_(bpy-SC)]^2+^ appears to be of great interest as a potential antimetastatic agent. Its preferential accumulation in a cellular cytoskeleton fraction, in which increased expression of MMP-9 was assessed, may point to matrix metalloproteinases as one of the possible targets. It must be noted that GM6001 although it is a good inhibitor of MMPs, in particularly MMP-9, it did not affect the adhesion properties of cells in the trypsin resistance assay. Moreover, NAMI-A which increased the fraction of adherent cells in trypsin resistant assay did not affect MMPs activity. Therefore, protease inhibition is an important feature of ruthenium(II) polypyridyl complexes, in particular for [Ru(dip)_2_(bpy-SC)]^2+^; however other molecular targets need to be identified to better understand its effect on cell adhesion. 

## 3. Materials and Methods

### 3.1. Materials

All solvents were of at least analytical grade and were used without further purification. Unless otherwise stated, reagents were purchased from Sigma-Aldrich (St. Luis, MO, USA). All aqueous solutions were prepared with deionized MiliQ-class water from Millipore (Burlington, MA, USA) system. The following complexes [Ru(dip)_2_(bpy)]Cl_2_, [Ru(dip)_2_(bpy-SC)]Cl_2,_ [Ru(dip)_3_]Cl_2_ and NAMI-A were prepared according to the published procedures [13,24]. The detailed synthesis of [Ru(dip)_2_(dpb)]Cl_2_, [Ru(dip)_2_(dpq)]Cl_2_ and [Ru(dip)_2_(dpq(CH_3_)_2_)]Cl_2_ is described in the submitted manuscript [30]. The stock solutions of Ru(II) polypyridyl complexes were prepared in DMSO while aqueous solution of NAMI-A was freshly prepared before each experiment and kept on ice no longer than 30 min. Lipophilicity (clogP) of all used ligands was calculated with the use of ChemDraw Professional 17.1 (Perkin Elmer, Waltham, MA, USA).

### 3.2. Cell Culture and Cytotoxicity Assay

In vitro studies were conducted using human lung adenocarcinoma A549 cell line. Cells were routinely cultured in Dulbecco’s Modified Eagle Medium (DMEM, Corning, Corning, NY, USA) supplemented with 10% fetal bovine serum (FBS, Eurx, Gdańsk, Poland) (*v*/*v*) and 1% penicillin-streptomycin (100 units/mL–100µg/mL, Corning) (*v*/*v*) and incubated at 37 °C in humidified atmosphere with 5% CO_2_ (*v*/*v*). The cytotoxicity of the Ru(II) complexes was evaluated using MTT assay (Sigma-Aldrich). Cells were seeded into 96-well plate with the density of 3 × 10^4^ cells per cm^2^ in complete medium and cultured for 24 h. Then medium was removed and various concentrations of the studied compounds in basic medium were added. The final DMSO concentration in cell culture was fixed at 0.1% (*v*/*v*). After 24 h of incubation with Ru(II) complexes in the concentration range 0–32 µM, cells were washed with medium and 100 µL of MTT solution (0.5 mg/mL) was added for 3 h. NAMI-A was evaluated up to 200 µM concentration. After the incubation, MTT was removed, and the formed violet formazan crystals were dissolved in the 100 µL of DMSO:methanol (1:1) mixture. The absorbance was measured using Tecan Infinite 200 microplate reader (Tecan, Männedorf, Switzerland) at 565 nm with 700 nm as a reference wavelength. Experiments were performed in triplicates and repeated three times. IC_50_ parameters were determined using Hill equation (OriginPro 2020, OriginLab Corporation, Northampton, MA, USA) and are presented as mean values and the standard deviation of the mean.

### 3.3. Cellular Uptake

For cellular uptake A549 cells were seeded in a 6-well plate with the density of 4 × 10^4^ cells per cm^2^ in complete medium and cultured for 24 h. Then the medium was removed, and cells were treated with 1 µM (non-toxic concentration) of all studied Ru(II) complexes in a basic medium for 24 h. Any dead cells were eliminated by washing cells’ monolayer with PBS. Subsequently, the incubated cells were washed, detached by trypsin treatment and counted. The cells were isolated by centrifugation and digested in concentrated nitric acid overnight at room temperature. The solutions were diluted with Millipore water to a final nitric acid concentration of 2%. The Ru content in the samples was measured by inductively coupled plasma mass spectrometry (ICP-MS) using NexION 2000C (Perkin Elmer). Experiments were repeated three times. Results were calculated as ruthenium concentration per cell and presented as mean values and standard deviation of mean. Similarly, the content of Ru ions was measured in subcellular fraction obtained as described in chapter 4.5.

### 3.4. Trypsin Resistance Assay

Cells susceptibility to detachment upon incubation with the studied compounds was evaluated by checking their resistance to trypsin treatment. Cells were seeded into 96-well plate with the density of 3 × 10^4^ cells per cm^2^ in complete medium and cultured for 24 h. Then, cells were incubated with various concentrations of the studied compounds for 24 h. Subsequently, the cells were washed and 30 µL trypsin solution (0.05%) was added to each well for 10 min incubation at 37 °C. Next, cells were washed with PBS and Alamar Blue assay was performed to quantify adherent cells. The received results were normalized with appropriate wells without the trypsin treatment to exclude the possible toxicity of the studied compounds and presented as a percentage of control (untreated) cells. Experiments were performed in triplicates and each experiment was repeated five times to obtain mean values and standard error of the mean.

### 3.5. Subcellular Fractionation

Subcellular fractionation was performed using the subcellular protein fractionation kit for cultured cells (Thermo Fisher Scientific, Waltham, MA, USA) according to the manufacturer’s instructions. Briefly, A549 cells were seeded in a 25 cm^2^ flask with the density of 1.25 × 10^6^ cells per flask in complete medium and cultured for 24 h. Then, the medium was removed, and cells were treated with 4 µM (non-toxic concentration) of [Ru(dip)_2_(bpy-SC)]Cl_2_ or [Ru(dip)_2_(dpq)]Cl_2_ in a basic medium for 24 h. After the incubation cells were washed with PBS, detached by trypsin treatment, and counted. A total of 2 mln cells were collected for fractionation in 1.5 mL microcentrifuge tubes. The following fractions were obtained: CEB—cytoplasmic, MEB—membrane, NEB1—soluble nuclear, NEB2—chromatin bound nuclear, PEB—cytoskeletal. The prepared extracts were digested in a concentrated nitric acid overnight at 60 °C and later analyzed by inductively coupled plasma mass spectrometry (ICP-MS) using NexION 2000C (Perkin Elmer). Experiments were repeated three times. Results were calculated as percentage of ruthenium concentration per cell and presented as mean values and standard deviation of mean. Additional samples were prepared and used for quantitative analysis of MMP-2/9 using Western blot as described in chapter 4.6.

### 3.6. Western Blot Analysis

A549 cells were seeded in a 25 cm^2^ flask with the density of 1.25 × 10^6^ cells per flask in complete medium and cultured for 24 h. Then the medium was removed, and cells were treated with 1 or 4 µM (non-toxic concentration) of Ru complexes in a basic medium for 24 h. After the incubation, cells were washed with ice-cold PBS and lysed using RIPA buffer containing Halt proteases inhibitor cocktail (Thermo Fisher Scientific). Protein samples (the same concentration per lane) were separated on a 12% sodium dodecyl sulfate polyacrylamide gel. PageRuler™ Prestained Protein Ladder (Thermo Fisher Scientific) was used to determine approximate molecular weights of resolved proteins. Electrophoresis was performed through the stacking gel for 25 min at 60 V and resolving gel for 50 min at 170 V at room temperature using a PowerPac™ Basic Power Supply (Bio-Rad, Inc., Hercules, CA, USA). A wet electrotransfer was carried out for 2 h at a constant current of 200 mA to transfer the separated proteins to a polyvinylidene difluoride (PVDF) membrane (Bio-Rad, Inc., Hercules, CA, USA), followed by blocking in 5% skim milk in TBST over 1 h (Tris-buffered Saline Tween-20). Furthermore, the membrane was probed with primary antibody overnight at 4 °C: mouse monoclonal anti-MMP-2 antibody (dilution 1:200; Thermo Fisher Scientific, catalog number MA5-13590); rabbit polyclonal anti-MMP-9 antibody (dilution 1:2000; Thermo Fisher Scientific, catalog number PA5-13199) or mouse monoclonal anti-β-actin antibody (dilution 1:1000; Thermo Fisher Scientific, catalog number AM4302). After being washed three times in TBST membrane was incubated with polyclonal secondary antibody conjugated with horseradish peroxidase for 2 h in room temperature: goat anti-mouse antibody (dilution 1:10,000; Thermo Fisher Scientific, catalog number G21040) for MMP-2/β-actin detection or goat anti-rabbit antibody (dilution 1:10,000; Thermo Fisher Scientific, catalog number G21234) for MMP-9 detection. Reactive protein was detected after membrane was washed three times in TBST using GE Healthcare Amersham™ ECL Prime Western Blotting Detection Reagent (GE Healthcare Inc., Chicago, IL, USA). Only one protein was detected at a time, each membrane was stripped in 0.2 M NaOH over 20 min, washed three times in water and blocked again before proceeding with another primary antibody. Data were collected by ChemiDoc XRS+ Imaging System (Bio-Rad, Inc., Hercules, CA, USA) and analyzed with Image Lab v. 6.1.0 software (Bio-Rad, Inc., Hercules, CA, USA). β-actin was used for normalization. Active form of MMP-9 was identified at 70 kDa.

### 3.7. MMP-2 and MMP-9 Inhibition

Pro-enzymes (MMP-2 and MMP-9), fluorogenic substrate FS-6 (Mca-Lys-Pro-Leu-Gly-Leu-Dpa-Ala-Arg-NH_2_) and reference inhibitor for MMPs GM6001 were obtained from Sigma-Aldrich. MMP-9 and MMP-2 were activated at 4 °C using 1 mM APMA overnight before the experiments. MMP-2 and MMP-9 assays were performed in 0.1 M Tris/HCl (pH 7.4, 37 °C) enriched with 0.1 M NaCl, 10 mM CaCl_2_, 0.1 mM ZnCl_2_ and 0.05% Brij35. The final concentrations of the enzyme and substrate (FS-6) were kept constant at 0.4 nM and 2.5 µM, respectively. Kinetic fluorescence measurements were performed using a Perkin Elmer LS55 spectrofluorimeter (*λ_ex_*/*λ_em_* = 325/400 nm). The obtained fluorescence intensities were corrected due to inner filter effect according to the following equation [31]:

$$F_{corr}=F_{obs}\cdot 10^{A_{\lambda em}+A_{\lambda ex}}$$

where *F_obs_* denotes the measured fluorescence intensity, while *A_λem_* and *A_λex_* are the absorbance of sample at emission and excitation wavelengths. 

The enzyme activity in the presence of inhibitors was expressed as a fraction of initial reaction rate (v_i_/v_0_ ratio, v_0_—initial reaction rate, v_i_—initial reaction rate in the presence of inhibitor). IC_50_ parameters were calculated from a dose-response plot of enzyme fractional activity as a function of inhibitor concentration, using the Hill equation (OriginPro2018). Experiments were repeated three times and results are presented as mean values and standard deviation of the mean.

To confirm that the changes in fluorescence of FS-6 are solely attributed to the enzymatic activity of the MMPs and are not interfered by the possible quenching effect of Ru(II) polypyridyl compounds, the formation of the fluorescent product resulting from the cleavage of FS-6 was followed by HPLC. Reaction between MMP-9 and FS-6 was carried out under the same buffered conditions as for the spectrofluorimetric measurement. After 1, 2, 3, 4, and 5 min 100 µL of the reaction mixture was taken and diluted 10 times with GM6001 solution, a commercially available MMP inhibitor with an IC50 of 0.6 µM (the final concentration of GM6001 was 8 µM) for blocking MMP-9 activity. Samples were placed on ice and separated by HPLC as soon as possible. The chromatograms were registered using a Perkin Elmer HPLC Chromera system equipped with fluorescence detector (λ_ext_ 325 nm, λ_em_ 400 nm). Separation was obtained on Brownlee Bio C18 150 × 4.6 mm column, with ammonia acetate (0.1 M, pH 4.6) and acetonitrile as solvents applied for gradient elution from 5 to 40% of acetonitrile for 20 min with a flow rate of 1 mL/min. The inhibition activity was determined for [Ru(dip)_2_(bpy-SC)]^2+^ applying the following concentrations: 0; 0.25; 1.25; 7.5; 12.5; 25 µM. The concentrations of MMP-9 and FS-6 were constant, 0.4 nM and 2.5 µM, respectively. Reaction between MMP-9 and FS-6 was carried out in the presence of each concentration of Ru complex and the progress of the reaction was monitored by the observation of the peak from the formed fluorescent product. The initial velocities were calculated from the peak areas and used for calculation of IC_50_ applying Hill model. IC_50_ determined for [Ru(dip)_2_(bpy-SC)]^2+^ from HPLC method was 4.8 ± 0.7 µM while from fluorescence assay 5.9 ± 1.0 µM. The spectrofluorimetric method is far more accurate than HPLC since it directly measures the signal without delay necessary in HPLC separation as well as stopping enzyme activity prior separation. Nevertheless, the obtained results point out that spectrofluorimetric assay can be successfully used for determination IC_50_ for Ru(II) polypyridyl complexes.

### 3.8. Statistical Analysis

Data are expressed as the mean ± standard error of the mean (SEM). Significant differences among tested samples were determined by one way analysis of variance (ANOVA) using Statistica 13 software. Probabilities of *p* < 0.05 were considered to be statistically significant. The following notification is used * *p* < 0.05.

## 4. Conclusions

Inhibiting metastasis at the very beginning is essential for successful anticancer therapy. Cell detachment from the primary tumor followed by invasion is considered the early phase of metastasis [32]. Among the studies series of Ru(II) polypyridyl complexes three of them namely [Ru(dip)_2_(bpy-SC)]^2+^, [Ru(dip)_2_(dpb)]^2+^ and [Ru(dip)_3_]^2+^ demonstrated high activity in inhibiting of cell detachment along with quite efficient inhibition of MMP-2 and MMP-9. More detailed studies for [Ru(dip)_2_(bpy-SC)]^2+^ showed its preferential accumulation in the cytoskeleton, where also intracellular MMP-9 was localized to a greater extent, pointing to this enzyme as one of the targets for this compound. Since GM6001, an excellent MMP-9 inhibitor, did not induce the enhancement of cell adhesion, it can be assumed that other targets, most likely localized in the cytoskeleton, are involved in the biological activity of these compounds. Current knowledge indicates that only those compounds that can act simultaneously on several targets in the metastatic cascade can successfully inhibit metastasis [33], therefore the studied compounds are good candidates for their application as antimetastatic agents. 

## Figures and Tables

**Table 1:** IC_50_ values for inhibition of MMP-2 and MMP-9 by studied compounds as well as the reference metalloproteinase inhibitor GM6001 and antimetastatic agent NAMI-A. Experimental conditions: [FS-6] = 2.5 µM, [enzyme] = 0.5 nM, 0.1 M Tris, pH 7.4 at 37 °C (MMP-2 and MMP-9 were activated using 1 mM APMA at 4 °C overnight, and the buffer was supplemented with 0.1 M NaCl, 10 mM CaCl_2_, 0.1 mM ZnCl_2_ and 0.05% Brij35).

Compound	IC_50_ for MMP-2 [µM]	IC_50_ for MMP-9 [µM]
[Ru(dip)_2_(dpq)]^2+^	n.i.	n.i.
[Ru(dip)_2_(dpq(CH_3_)_2_]^2+^	n.i.	16.8 ± 9.4
[Ru(dip)_2_(bpy)]^2+^	22.5 ± 2.0	6.2 ± 2.8
[Ru(dip)_3_]^2+^	8.3 ± 1.1	4.1 ± 1.6
[Ru(dip)_2_(dpb)]^2+^	13.1 ± 4.6	9.0 ± 3.7
[Ru(dip)_2_(bpy-SC)]^2+^	8.0 ± 1.1	5.9 ± 1.0
GM6001	34 ^1^	0.6 ^1^
NAMI-A	n.i.	n.i.

^1^ data taken from reference [19]. n.i.—no inhibition in the studied ranged (>> 25 µM, for NAMI-A >> 600 µM).

## Data Availability

The data presented in this study are available in the main text and the Supplementary Materials.

## Supplementary Materials

The following are available online at https://www.mdpi.com/article/10.3390/ph14101014/s1, Synthetic protocols, Figure S1: Western blot analysis.
