# Supplementary material for: Inhibition of Matrix Metalloproteinases and Cancer Cell Detachment by Ru(II) Polypyridyl Complexes Containing 4,7-Diphenyl-1,10-phenanthroline Ligands—New Candidates for Antimetastatic Agents
Source: Pharmaceuticals (Basel). 2021 Oct 1;14(10):1014. doi: 10.3390/ph14101014 (PMC8538513; doi:10.3390/ph14101014)
Supplement: Supplementary file 1 [file pharmaceuticals-14-01014-s001.zip › Supporting Pharmaceuticals MMPs Brindell (1).pdf]

Supporting information to

Inhibition of Matrix Metalloproteinases and Cancer Cell Detachment by Ru(II) Polypyridyl Complexes Containing 4,7-Diphenyl-1,10-Phenanthroline Ligands—New Candidates for Antimetastatic Agents

Przemysław Gajda-Morszewski [1], Ilona Gurgul [1], Ewelina Janczy-Cempa [1], Olga Mazuryk [1], Michał Łomzik[1,2] and Małgorzata Brindell [1,*]

[1] *Faculty of Chemistry, Jagiellonian University in Krakow, Gronostajowa 2, 30-387 Krakow, Poland; pgmorszewski@doctoral.uj.edu.pl, ilona.gurgul@doctoral.uj.edu.pl, ewelina.janczy@doctoral.uj.edu.pl, olga.mazuryk@uj.edu.pl, michal.lomzik@chemia.uni.lodz.pl*

[2] *Department of Organic Chemistry, Faculty of Chemistry, University of Łódź, ul. Tamka 12, 91-403 Łódź, Poland*

* Correspondence: malgorzata.brindell@uj.edu.pl

1. Synthetic protocols:

Synthesis of [Ru(dip)$_2$]Cl$_2$

Ru(dip)$_2$Cl$_2$ complex was obtained following the literature procedure [1]. Ruthenium(III) chloride trihydrate (1 mmol), 4,7-diphenyl-1,10-phenanthroline (2 mmol), and 2 drops of N-ethylmorpholine were dissolved in degassed N,N-dimethylformamide (10 mL) under argon. The mixture was refluxed for 24 h. Then the solvent was removed under reduced pressure to c.a. 1 mL and acetone (20 mL) was added. After 24 h at -20°C dark solid was filtrated, and washed with cold acetone, twice with diethyl ether, and dried by suction. The crude product was purified by flash chromatography (Al_2O_3 neutral, dichloromethane/methanol (98/2)) to give the pure product. The purity of the synthesized complex was confirmed by HPLC and MS analyses.

Synthesis of [Ru(dip)$_2$bpy]Cl$_2$ and [Ru(dip)$_2$(bpySC)]Cl$_2$

[Ru(dip)$_2$(bpySC)]Cl$_2$ was prepared according to the published procedures [2], while synthesis for [Ru(dip)$_2$(bpy)]Cl$_2$ was described in [1]. Briefly, to a solution of Ru(dip)$_2$Cl$_2$ (0.023 mmol) in absolute ethanol (10 mL) under argon conditions, solution of ligand (bpy or bpySC, 0.026 mmol) in absolute ethanol (5 mL) was added. Mixtures were refluxed for 24 hours. After that time, the solvent was removed under reduced pressure. The crude products were dissolved in dichloromethane and purified by flash chromatography (Al_2O_3 neutral, dichloromethane/methanol (95/5) to give pure products, the purity of which was confirmed by HPLC and MS analyses.

Synthesis of [Ru(dip)$_2$(dpq)]Cl$_2$, [Ru(dip)$_2$(dpb)]Cl$_2$ and [Ru(dip)$_2$(dpq-CH$_3$)]Cl$_2$

[Ru(dip)$_2$(dpq)]Cl$_2$, [Ru(dip)$_2$(dpb)]Cl$_2$ and [Ru(dip)$_2$(dpq-CH$_3$)]Cl$_2$ were synthesized according to the procedure described in the submitted manuscript [3]. 6,7-dimethyl-2,3-bis(2-pyridyl)quinoxaline (dpq(CH$_3$)$_2$) were purchased from Sigma-Aldrich while two other ligands 2,3-bis(2-pyridyl)quinoxaline (dpq) and 2,3-bis(2-pyridyl)benzo[g]quinoxaline (dpb) were obtained following the literature procedure [4]. Solution of Ru(dip)$_2$Cl$_2$ (0.05 mmol) in absolute ethanol (5 mL) was mixed with a solution of appropriate ligand L (dpq, dpq(CH$_3$)$_2$ or dpb (0.05 mmol)) in absolute ethanol (3 mL). The mixture was refluxed for 24 h. Then the solvent was removed under

reduced pressure and red-orange residues were dissolved in water (10 mL) and filtrated. The filtrate dissolved dichloromethane with a few drops of methanol and precipitated out with diethyl ether. The powder was washed with diethyl ether and dried to give the pure product. The purity and identity were confirmed by HPLC and HRMS.

Synthesis of [Ru(dip)$_3$]Cl$_2$

[Ru(dip)$_3$]Cl$_2$ was prepared following a modified literature procedure [5]. Ruthenium(III) chloride trihydrate (3 mmol) and 4,7-diphenyl-1,10-phenanthroline (17 mmol) were suspended in 150 mL of ethanol. After refluxing overnight, the mixture was cooled, and solvent was removed under reduced pressure. Crude product was purified by flash chromatography (Al_2O_3 neutral, acetonitrile (100%) ramped to 10% deionized H_2O and 1,5% KNO_3 in acetonitrile). The purity of the synthesized complex was confirmed by HPLC and MS analyses.

Synthesis of NAMI-A

NAMI-A (HIm)[*trans*-RuCl$_4$(dmso)(Im)] was previously prepared following the reported procedure [6]. Briefly, the precursor (H(dmso)$_2$)[*trans*-RuCl$_4$(dmso)$_2$] was synthesized by suspension of 0.5 g of RuCI$_3$ ×3 H_2O (0,0019 moles) in 30 ml ethanol followed by heating under reflux for 3 h to obtain a green solution. The solution was filtered and concentrated up to 1/10 of the initial volume. To the obtained solution 1 ml of HCI, 37% and 2 ml of DMSO were added and the mixture was kept at 80°C for 15 min. after which orange solution appeared. After cooling to room temp. 10 ml acetone and of a few drops of ethyl ether were added resulting in red-orange precipitation. The formed product was collected on the filter and wash with cold acetone and ethyl ether.. Furthermore, 0.5 g (0.0009 moles) of (H(dmso)$_2$)[*trans*-RuCl$_4$(dmso)$_2$] was suspended in 10 ml of acetone in room temp. and 0.245 g (0.0036 moles) of imidazole was added. The mixture was stirred for 4 h and the formed brick red precipitated was collected on the filter and wash with acetone (10 ml) and ethyl ether (10 ml). Its purity was confirmed by elementary analysis and UV–Vis spectroscopy.

2. Western Blot analysis

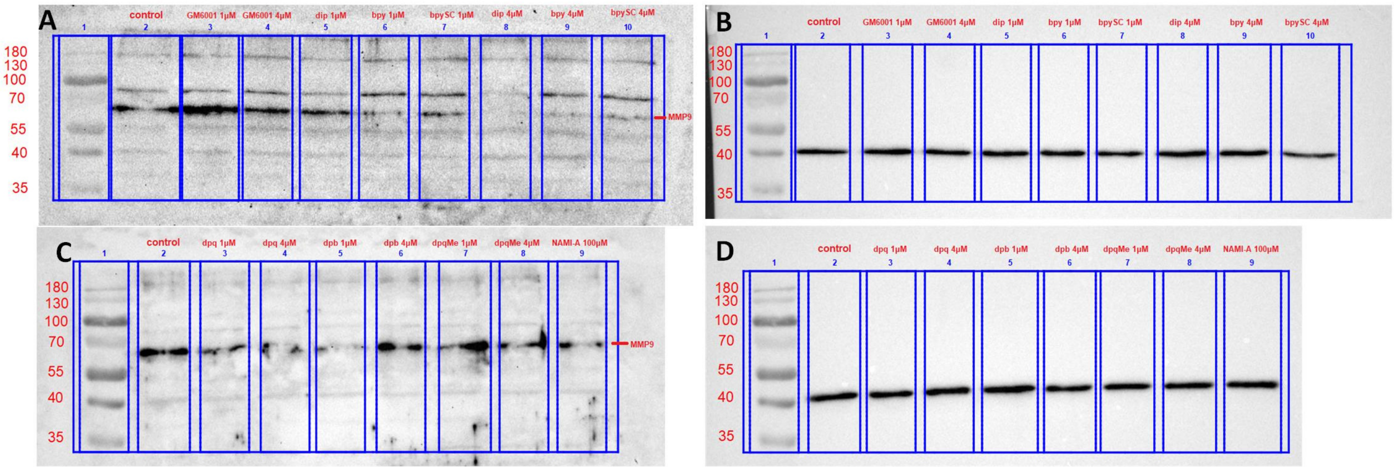

Figure S1. Representative images of Representative image of Western blot detection of MMP-9 activity (A, C) and β-actin (B, D) measured in A549 cells after 24 h incubation with 1 or 4 µM of Ru complexes or GM6001, and 100 µM of NAMI-A. β-actin was used for normalization.

References

1. Regis Caspar, †; Christine Cordier, ‡; Jenny B. Waern, †; Carine Guyard-Duhayon, †; Michel Gruselle, †; Pascal Le Floch, § and; Hani Amouri*, † A New Family of Mono- and Dicarboxylic Ruthenium Complexes [Ru(DIP)2(L2)]2+ (DIP = 4,7-Diphenyl-1,10-Phenanthroline): Synthesis, Solution Behavior, and X-Ray Molecular Structure of Trans-[Ru(DIP)2(MeOH)2][OTf]2. *Inorganic Chemistry* **2006**, *45*, 4071–4078, doi:10.1021/IC0601236.
2. Łomzik, M.; Mazuryk, O.; Rutkowska-Zbik, D.; Stochel, G.; Gros, P.C.; Brindell, M. New Ruthenium Compounds Bearing Semicarbazone 2-Formylopyridine Moiety: Playing with Auxiliary Ligands for Tuning the Mechanism of Biological Activity. *Journal of Inorganic Biochemistry* **2017**, *175*, 80–91, doi:10.1016/J.JINORGBIO.2017.07.006.
3. Mazuryk, O.; Janczy-Cempa, E.; Kuśnierz, J.; Rutkowska-Zbik, D.; Machnicka, A.; Stochel, G.; Brindell, M. Relevance of Electron Transfer Pathway in Photodynamic Activity of Ru(II) Polypyridyl Complexes Containing 4,7-Diphenyl-1,10-Phenantroline Ligands under Normoxic and Hypoxic Conditions . *Dalton Transactions (Under review)*.
4. Goodwin, H.A.; Lions, F. Tridentate Chelate Compounds. II1. *Journal of the American Chemical Society* **2002**, *81*, 6415–6422, doi:10.1021/JA01533A022.
5. Mabrouk, P.A.; Wrighton, M.S. Resonance Raman Spectroscopy of the Lowest Excited State of Derivatives of Tris(2,2′-Bipyridine)Ruthenium(II): Substituent Effects on Electron Localization in Mixed-Ligand Complexes. *Inorganic Chemistry* **1986**, *25*, 526–531, doi:10.1021/ic00224a027.
6. Mestroni, G.; Alessio, E.; Sava, G. Patent for Salts of Anionic Complexes of Ru(III), as Antimetastatic and Antineoplastic Agents; World Intellectual Property Organization WO 98/00431 1997.
